# Degradation Detection in a Redundant Sensor Architecture

**DOI:** 10.3390/s22124649

**Published:** 2022-06-20

**Authors:** Amer Kajmakovic, Konrad Diwold, Kay Römer, Jesus Pestana, Nermin Kajtazovic

**Affiliations:** 1 Pro2Future GmbH, 8010 Graz, Austria; konrad.diwold@pro2future.at (K.D.); jesus.pestana@pro2future.at (J.P.); 2 Institute of Technical Informatics, Graz University of Technology, 8010 Graz, Austria; 3 Siemens AG, 8054 Graz, Austria; nermin.kajtazovic@siemens.com

**Keywords:** degradation, drift, discrepancy, redundant sensors, 1oo2 architecture

## Abstract

Safety-critical automation often requires redundancy to enable reliable system operation. In the context of integrating sensors into such systems, the one-out-of-two (1oo2) sensor architecture is one of the common used methods used to ensure the reliability and traceability of sensor readings. In taking such an approach, readings from two redundant sensors are continuously checked and compared. As soon as the discrepancy between two redundant lines deviates by a certain threshold, the 1oo2 voter (comparator) assumes that there is a fault in the system and immediately activates the safe state. In this work, we propose a novel fault prognosis algorithm based on the discrepancy signal. We analyzed the discrepancy changes in the 1oo2 sensor configuration caused by degradation processes. Several publicly available databases were checked, and the discrepancy between redundant sensors was analyzed. An initial analysis showed that the discrepancy between sensor values changes (increases or decreases) over time. To detect an increase or decrease in discrepancy data, two trend detection methods are suggested, and the evaluation of their performance is presented. Moreover, several models were trained on the discrepancy data. The models were then compared to determine which of the models can be best used to describe the dynamics of the discrepancy changes. In addition, the best-fitting models were used to predict the future behavior of the discrepancy and to detect if, and when, the discrepancy in sensor readings will reach a critical point. Based on the prediction of the failure date, the customer can schedule the maintenance system accordingly and prevent its entry into the safe state—or being shut down.

## 1. Introduction

Sensors are an indispensable part of industrial automation systems, as they allow important environmental information such as light, heat, motion, or pressure to be evaluated and integrated into the automation process. As the number of sensors and sensor types increases, so does the impact of sensor failures on an automation system. Automation requires trustworthy sensors, because they are at the beginning of the control chain, and any incorrect information can lead to serious failures in the process. The effects of sensor failures can propagate throughout the entire system and cause further damage to machines or even workers. In a safety-critical system, sensors must be fail-safe [1]. This means that any type of instability must be detected and acted upon to prevent catastrophic failures, indicating that the information they provide must be reliable. The reliability of the sensor can be increased by using high-quality and safety-certified sensors, which normally have higher costs. Alternatively, multiple non-safety-certified sensors can be configured and used in one of the redundant safety architectures [2].

The rising complexity of the automation systems, particularly sensor systems, increased the number of sensor fault occurrences, consequently reducing reliability, safety, and, in the end, availability parameters of the system. In general, there are many causes for faults occurrence in sensor systems, but the most common are process variation, ongoing wear-out, natural aging process, mechanical defects, loose connection, and harsh environmental conditions.

According to Chenyu Xiao et al. [3], faults in sensors systems can be classified into permanent and intermittent faults. Permanent faults (e.g., abrupt faults and incipient faults) will not resolve once they occur, while intermittent faults only last for a limited period of time, after which the functionality is restored without any repairing maintenance activities [4]. Intermittent faults can easily evolve into permanent faults, thus leading to system disruption. As intermittent faults can often be viewed as a prelude to permanent faults, they are subject to recent research in fault detection and prognosis [5,6].

**Figure 1:**
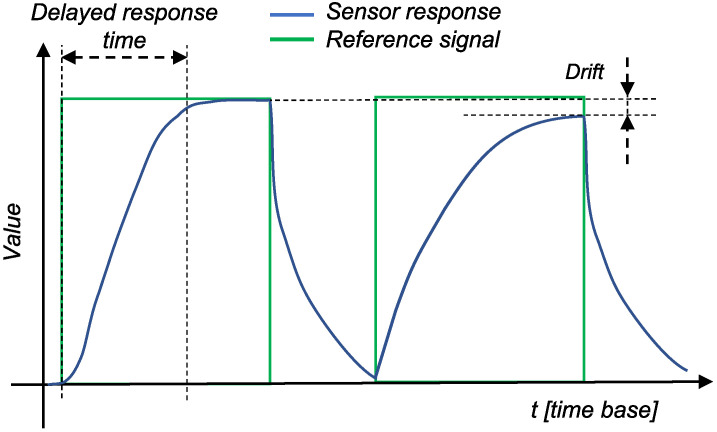
Drift and delay or sensor response.

During its lifetime, a digital sensor is exposed to various stresses in addition to an aging process. Therefore, wear- and aging-related faults are very common [7]. The two most common causes of sensor aging-related failures are changes in sensor calibration and changes in response time [8]. Calibration drift (shift) can be caused either by various mechanical problems or by the leakage or degradation of the filling fluid in the sensor itself. Figure 1 shows the general problems related to sensor measurements, with the green line representing the reference signal (changes in the measurement medium) and the blue line representing the sensor response. In the second response in Figure 1, the baseline response of the sensor is clearly shifted. Therefore, the sensor will never reach the actual value of the measured stimulus. In the specific case of gas sensors, for example, these drifts are due to dynamic processes in the sensor system (e.g., poisoning or aging of the sensors) or to environmental changes (e.g., temperature and pressure conditions), and drift is mostly described with a linear line [9]. Several studies [10,11,12] have analyzed the drift of pressure sensors. All studies have shown that the drift in the pressure sensor varies mostly linearly or, in rare cases, exponentially. Similar studies for gas [13] and pH [14] sensors had successfully modeled the drift using the exponential and linear laws, respectively. Linear drift is also found in the silicon humidity sensor, as shown in [15]. Nevertheless, the sensors drift of the same type can be described by the same model, but with different parameters of the model. Problems with response time, on the other hand, are usually due to problems in the electronics of a sensor. The first response in Figure 1 represents a delay, indicating that the sensor did not respond immediately to changes in the measured medium. Sensor aging and its modeling is a highly active research topic (see Section 2 for more details).

The faults caused by aging and wear can be permanent or intermittent. Permanent faults can be detected relatively easy, while intermittent faults are more dangerous for the sensor system because they mostly occur in irregular intervals and are hard to detect. Sensors may exhibit a drift or delay during certain periods of time (i.e., when an intermittent fault occurs), while functioning normally at other times.

In addition to the aging and wear problems, sensors can also experience wiring problems such as short circuits or wire breaks. Wire breaks can be easily detected by using a spatial safety signal or special hardware architectures that can be integrated to detect wire-related problems on the sensor side [16,17]. Therefore, special input/output modules have recently been developed to detect such problems in sensors. These modules are usually named with a prefix “fail-safe” [18].

Since the drift of a single sensor changes over time, the following question arises: What are the consequences of such drift for a system when a redundant architecture is used (e.g., 1oo2)? Such systems compare and validate the redundant sensor readings in order to ensure correct system behavior. One measure of a comparison is called discrepancy analysis, which is used to compare readings from the redundant sensors. It is performed by subtracting the sensor readings and then comparing the resulting discrepancy value to the maximum value set by the user. If the discrepancy reaches the maximum value, this means that one of the sensors is providing incorrect readings, which is interpreted by the system as an error. Due to the unequal drifts in the redundant sensors, the discrepancy between the levels also tends to change over time and may eventually reach a maximum value, which is again interpreted as an error.

Once a discrepancy has been detected, the system is transferred into a safe state to prevent further damage. This reduces the availability of the system. To circumvent the transition to a safe state, this work was carried out to detect and predict drift-based failures in redundant sensor setups and predict the time remaining until the threshold of the allowable discrepancy is reached. In contrast to transitioning to a safe state, this information could be used to plan and perform preventive maintenance, thus preventing a system outage.

It should be noted that, in this work, we considered the problem from the point of view of an automation system and not from the point of view of a sensor. This means that the derived methods can potentially be used to detect and predict drift-based faults for different sensor modalities (i.e., modeling specific sensor modalities is out of the question). In addition, the developed methods must be computationally effective and training-free, so that they can be easily deployed in an automation system, which prevents the use of computationally (and training-wise) expensive and intensive ML approaches.

## 2. State of the Art

In the past, the maintenance of sensors systems relied on a great deal of manual work and cross-checking. Today, with the advancement of data analysis tools, new techniques such as predictive maintenance (PdM) [19] and predictive fail-safe (PdF) [2] have emerged. While the PdM is a general technique used with industrial equipment, PdF is used more frequently to analyze the safety-related data generated by fail-safe systems. Both techniques use data from the industrial environment and equipment to detect and predict anomalies and potential defects in the system [2].

### 2.1. Sensor Fault Diagnosis

Two main approaches are used to provide reliable sensor performance: analytical approaches and hardware approaches.

#### 2.1.1. Analytical Approaches

Analytical approaches can be classified as statistical, artificial intelligence, model-based approaches, and their hybrids [7,20]. Model-based approaches are approaches that rely on models created based on the past experience and historical data of the modeled object. To detect anomalies in the sensors, the actual sensor behavior is compared to the sensor behavior estimated by the model. However, this method requires a significant amount of prior knowledge about the dynamics and behavior of the sensors and the plant in order to detect sensor degradation and to separate this from the degradation of the measured plant [21].

Advancements in data analytics, and particularly machine learning and neural networks, have enabled the widespread application of artificial intelligence in sensors to detect anomalies in their behavior before they fail to deliver trustworthy data. The drift present in sensors is an important problem that impairs the reliability, especially in gas chemical sensors that measure the presence of substances in the environment. Zhao et al. [22] integrated the improved long short-term memory (LSTM) to build a classifier model to overcome drift issues in gas sensors. Other researchers [21] applied the ARMAX model (a generalization of the autoregressive moving average (ARMA) model) to the dynamic and static properties of the sensor to detect sensor degradation. An example of statistical approaches can be found in [23], where the authors used statistical approaches to monitor sensor data for smart structural health monitoring (SHM) systems.

#### 2.1.2. Hardware Approaches

Hardware approaches are taken to examine redundancy, whereby the same physical phenomenon is measured with multiple sensors. Multiple measurements from different sensors enable the detection of the faulty sensor either by means of a voting algorithm or by conducting correlation analysis [21].

**Figure 2:**
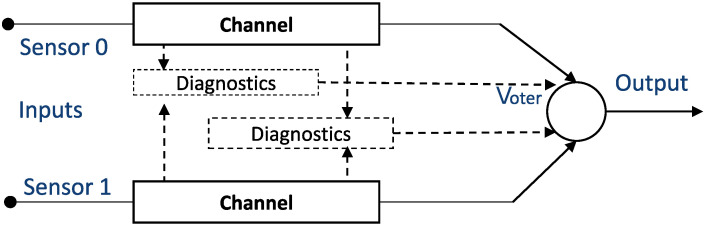
Model of the 1oo2D (1-out-of-2 with diagnostics) safety architecture.

Different redundant safety architectures are used for sensor diagnosis, such as 1oo2 (one-out-of-two), 1oo2D (one-out-of-two with diagnostics), or 2oo3 (two-out-of-three). To achieve a good balance between functional safety (i.e., achieving a high level of safety) and the cost of the sensor system, the most common strategy used with safety systems is to use a 1oo2 or 1oo2D architecture (see Figure 2), where hardware, including sensor inputs and software, are independently implemented twice [24].

In the context of sensors, this means that the parallel channels are checked and compared. If the signals from the sensors differ by a certain amount, the fail-safe mechanism will identify this as a failure and further passivate the channel or module. Therefore, the system is degraded, or, in the worst case, stopped completely, reducing the availability of the entire system.

An extended version of this method that incorporates the time characteristics of two redundant signals is called discrepancy analysis [17]. This analysis is initiated when different levels (i.e., drifts) are detected in two associated input signals. An analysis is made to determine whether the difference has disappeared after a maximum allowable period of time, which is known as the discrepancy time. By introducing discrepancy time, the algorithm has also become robust to the outliers. Discrepancy analysis is a standard in sensor diagnostics in fail-safe input devices [17,25,26] as well as in dual sensors [27]. Furthermore, its implementation is recommended in safety standards such as the ISO26262 and IEC 61508-6 [28,29]. Discrepancy analysis is introduced for both digital and analog sensor types and can be used to detect various problems that may occur in sensors, such as broken wires, short circuits, and mechanical and installation problems, as well as degradation.

### 2.2. Sensor Fault Prognosis

Fault prognosis is primarily used to estimate the remaining useful lifetime of a machine or component (e.g., a sensor) after an impending failure condition has been detected and identified [30]. A variety of techniques exist for fault prediction, ranging from Bayesian estimation and other probabilistic/statistical methods to artificial intelligence tools and methods based on computational intelligence terms [31,32]. According to Vachtsevanos et al. [31], fault prognosis prediction techniques can be divided into three categories: model-based, probability-based, and data-driven. Similar to fault diagnosis, model-based prediction techniques rely on dynamic models based on past experience and historical data. Prognosis models range from ARIMA family models to complex neural network models. For example, Wei et al. [33] applied the ARIMA approach to sensor data to predict the future of a rotor test rig, which could be used to simulate the operating condition of many rotating machines, such as gas turbines, compressors, and pumps. On the other hand, Wu at el. [34] used a more complex recurrent neural network (RNN) to predict the error in a dataset that contained data from different engines of the same type.

### 2.3. Long-Range Time Series Prediction

Since the dataset used in this work is a time series, a brief overview of techniques used to predicting long-term time series is presented below.

To describe the behavior of the discrepancy between sensors, an appropriate method for predicting long-term time series is required. Two groups of methods can be found in the literature: the classical time series prediction methods, such as linear regression (LR) [35], exponential regression (ER) [36], simple exponential smoothing (SES) [37], or autoregressive integrated moving average (ARIMA) methods [38], as well as machine learning (ML) methods [39]. Although ML methods promise better performance, there are a number of studies demonstrating that classical forecasting methods have outperformed complex machine learning methods. For example, Spyros Makridakis at el. showed in their 2018 study [40] that statistical models generally outperform ML methods across all forecast horizons, and especially for long-range forecasts.

Moreover, a difference in the performances of the classical time series prediction methods can be observed. The reports from the M3 [41] and M4 [42] competitions, where the forecasting methods were tested on several datasets from a variety of industries with different time intervals, show that simple methods (e.g., simple exponential smoothing or Holt–Winters methods) developed by practitioners perform as well as, or, in many cases, better than, statistically sophisticated models such as ARIMA and ARARMA [43].

## 3. Problem Statement, Research Contributions, and Method Overview

As described in Section 1, the responsiveness of a sensor decreases over its lifetime, i.e., it becomes “more sluggish”. This phenomenon is not easy to detect, because it is difficult to distinguish whether this is due to sensor aging or to the dynamics associated with the measured physical quantities. Sensor aging is not a uniform process; instead, it depends heavily on physical and environmental stressors to which the sensor is subjected. The manufacturing process can also play an important role for some sensors. This means that the probability of identical sensors aging with exactly the same dynamics is low, even if they are consistently exposed to the same load.

**Figure 3:**
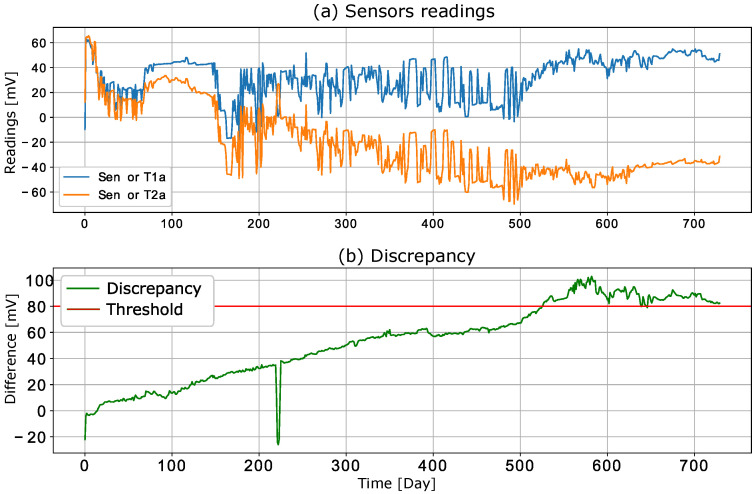
Data generated from the publicly available dataset [14]: (**a**) Sensor measurements averaged per day for two sensors; (**b**) discrepancy signal.

If a discrepancy analysis is conducted on a redundant sensor system, the delay time and drift between sensor readings can be expected to increase or decrease. Figure 3a shows the responses of two sensors (raw data from the public dataset [14]) that were exposed to the same chemical stress over a 730-day period. The discrepancy signal (i.e., the difference between the two sensor readings) is shown in Figure 3b, where the increase over time is evident. This means that a discrepancy between the sensors can reach a certain threshold that is considered a failure. This situation is also illustrated in Figure 3b, where the discrepancy reaches the threshold value of 25 (mV) on the 528th day of the experiment. Since this increase occurs over time, we may ask whether this process can be explained and whether this event can be predicted.

### 3.1. Research Contributions

The contribution of this paper is a novel fault prognosis algorithm [30,31,32] based on the discrepancy signal for the 1oo2 redundant sensor safety architecture. In our opinion, research on discrepancy analysis has been hampered by the lack of publicly available datasets containing data from redundant safety architectures (see Section 4) (e.g., 1oo2 and 2oo3). In comparison to classical discrepancy analysis [17,25,26,27], we first took model-based approaches [41,42] to predict the time at which the discrepancy signal would reach the defined threshold (Section 5.5); and, second, instead of using a time delay for the fail-safe trigger, we used a Hampel filter [44,45] to reject outliers (Section 6.5). These are significant improvements over classical discrepancy analysis, as our approach allows us to predict the date of failure before it occurs. Other authors performing work in the field of sensor arrays [46,47,48] have proposed methods that can be used to detect and compensate for drift between sensors. However, the impact of these methods on redundant sensor systems, and particularly their applicability to 1oo2 and 1oo2D safety architectures, have not yet been analyzed.

### 3.2. Method Overview

The article is divided into three parts. The first part reports the analysis of datasets, conducted to find suitable data for our approach. Section 4 presents the analyses of several publicly available databases containing measurements from redundant sensors that could be used to validate the degradation detection algorithm presented here. Based on these analysis results, the pH sensor dataset [14] was selected for a detailed analysis because it is the most complete dataset (i.e., long experimental duration, high sampling frequency). We started the analysis by applying the two most common trend-detection methods to detect increases in, decreases in, or the steadiness of the discrepancy. The results are presented in Section 4.

The second part of the article is reserved for determining the best-fitting discrepancy models (see Section 5). Several models were trained using the selected dataset [49] and their performance was compared. For each trained model, the root mean square error (RMSE) between the observed and fitted values was used for model comparison. This allowed the identification of the best-fitting models, which were sequentially selected for further use in the predictive applications presented in this study.

**Figure 4:**
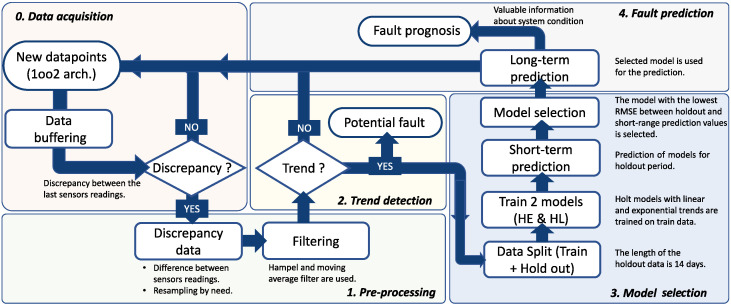
Flowchart outlining the steps in the proposed approach.

**Figure 5:**
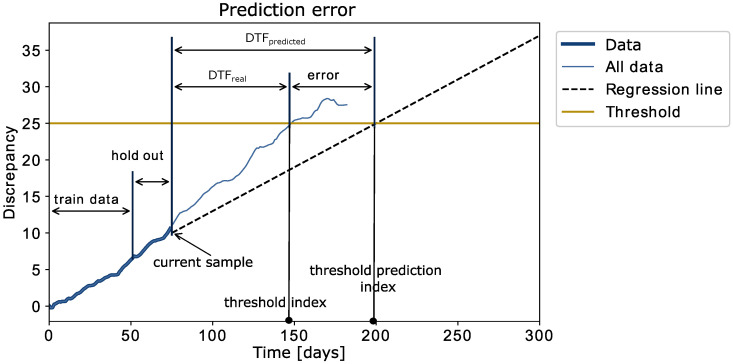
Explanatory figure of the prediction error calculations.

In the third part of the article, the steps of the proposed algorithm for detection and prediction of failure events are presented. In Figure 4, a flowchart of the algorithm is illustrated. The key requirement is that the targeted system must use a 1oo2 sensor architecture with discrepancy analysis (i.e., comparison) between the last sensor measurements. In addition, the system must provide adequate buffering of data that can be used later for analysis. This step is shown in the flowchart (Figure 4) as the initial, zero step. The first step is a preprocessing of the data. Since the data may display various anomalies, such as outliers, missing data points, or high-frequency noise that may affect the modeling, a preprocessing step is required to prepare the data for further analysis. In this step, two-step filtering is performed to clean the data and capture the main trend of the data. The filters and the type of filtering are explained in Section 6.5. Part of the data preprocessing may involve resampling the data series at a lower frequency. If the sampling frequency is too high, and we do not need so many data points, the data can be resampled to use only hourly, daily, or monthly average points. To determine whether the discrepancy changes at all, in the second step, trend detection techniques are used to detect an increase in, decrease in, or the steadiness of the discrepancy curve (see Section 6.4). This check step does not have to be performed each time a new data sample arrives, but can be defined by the number of samples (e.g., after every 100th sample) or over a period of time (e.g., daily, weekly, or monthly). If such a trend is not detected, data buffering continues until the next check step, when a trend detection method is run again. The detection of the trend in discrepancy data makes us doubt the reliability of the sensors. It also suggests that the sensors are not in the best condition, which increases the likelihood of failure. Therefore, the sensor system may need maintenance services, such as calibration or replacement. If a trend is detected in the data, the third step constitutes a model selection step. The buffered discrepancy data are split into training and holdout samples. The training samples are used to train two different forecasting models (i.e., Holt’s linear models), which are selected from a wider set of models based on the analysis presented in Section 5. After training, both models are used to perform a short-term prediction for the period of holdout values (14 days). The root mean square error (RMSE) between predicted and holdout values is calculated for both models and compared. The model with the lowest RMSE is selected for a long-range failure event prediction. A detailed explanation and results of the selection and prediction process can be found in Section 7, and a diagram outlining the process is shown in Figure 5.

If a trend is detected, a final fourth step is triggered. In this step, the best-fitting model (established in the previous step) is selected for prediction and is used to predict whether a fault event will occur and how much time remains before fault occurrence.

## 4. Dataset Selection

The investigation of discrepancy behavior requires a dataset with redundant sensors. Since aging behavior takes time to become apparent, and setting up a system to record sensor data in a controlled environment is a non-trivial and time-consuming task, we decided to use publicly available datasets. Therefore, the first step was to analyze available public datasets with redundant sensor data to identify suitable time series for our study.

**Figure 6:**
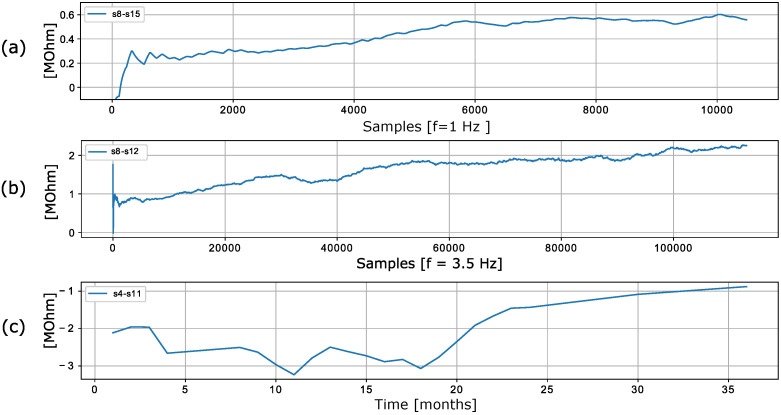
Discrepancy of (**a**) redundant MOX gas sensors presented in [50], (**b**) redundant gas sensors [51], and (**c**) redundant chemical sensors presented in [46].

The dataset used in the work [50] contains the time series of 16 chemical sensors exposed to gas mixtures at different concentrations. In particular, two gas mixtures are generated: ethylene and methane in air and ethylene and carbon monoxide (CO) in air. Although the dataset spans only a recorded duration of 12 h, an increase in the discrepancy of values is evident in the data. Figure 6a shows examples of discrepancies between two randomly selected sensors.

The dataset used in the work [51] was recorded using a chemical detection platform consisting of 14 temperature-modulated metal oxide semiconductor (MOX) gas sensors (seven units of TGS 3870-A04 and seven units of SB-500-12). The platform was exposed to dynamic mixtures of CO and humid synthetic air in a gas chamber. Drift between identical sensors is observed in the recorded data. Figure 6b shows an exemplary discrepancy between two sensors. The discrepancies between the sensors clearly change even though the sensors are of the same type and were exposed to the same stimuli.

The data presented in [46] contain measurements from 16 chemical sensors organized into four groups of four identical sensors (TGS2600, TGS2602, TGS2610, TGS2620). The authors used the sensors in drift compensation simulations to perform a discrimination task with six gases at different concentrations [46]. The data are shown in Figure 6c, which shows that the discrepancy between the sensors is not constant and changes over time.

Another interesting dataset is presented in [14]. The dataset represents a long-term study of the aging of ten individual pH sensors divided into two rows of five pairs of identical sensors, of which data from six sensors (T1a, T1b, T2a, T2b, T3a, T3b) are used. The rest of the sensors were destroyed during the experimental period and replaced with a different type of sensor, so long-term measurements are not available. The experiment lasted 731 days and samples were taken at a frequency of 1 Hz. The pH sensors were exposed to nitrified urine. At regular intervals (47 days in total), the sensors were removed from their normal position and exposed to other calibration media to characterize the sensors (drift and sensitivity) [14]. In this procedure, authors applied a two-point calibration procedure where the manufacturer’s recommended calibration solutions (with 7.00 pH and 4.01 pH) were used along with water to measure the drift and sensitivity of the sensors. The detailed procedure is described in [14].

In comparison to other identified datasets listed in Table 1, the last one [14] has the most complete information and the longest duration. Some of the other datasets contained only partial data or discontinuous data [52] or the duration of the collected data was not long enough to be used for modeling [53,54]. Moreover, some of the studies did not include detailed timestamps [52] for data points or the timestamps were relative to the start of the measurements [55], so continuity was lost. In addition, technical problems were experienced with all presented datasets during the experiment, so some data points were lost.

Unlike the other datasets found in the literature, the pH dataset [14] provides information about the drift and sensitivity of the sensors acquired during the calibration process. This dataset had a strictly defined change in the measurement medium, whereas the experiments from the other datasets were performed without a specific order, and the medium changed almost randomly. When all properties are considered, the collected data seem to be reliable and the best choice for a more in-depth analysis.

**Figure 7:**
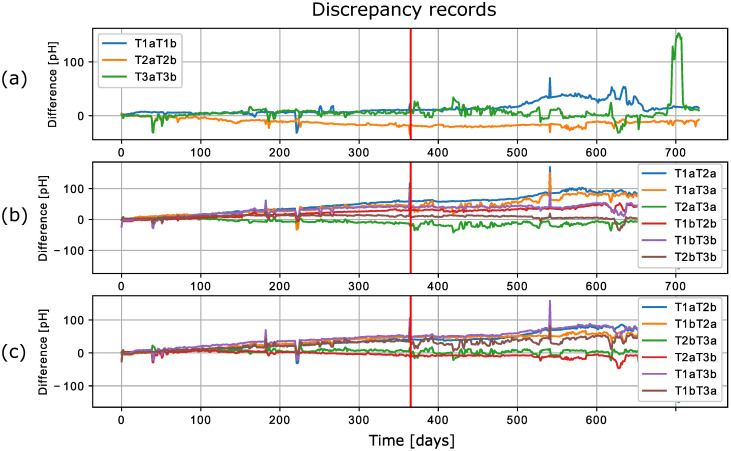
Discrepancy data for six pH sensors presented in [14], where graph (**a**) shows discrepancies of the identical sensors, and (**b**,**c**) show discrepancies among the rest of the sensors.

**Figure 8:**
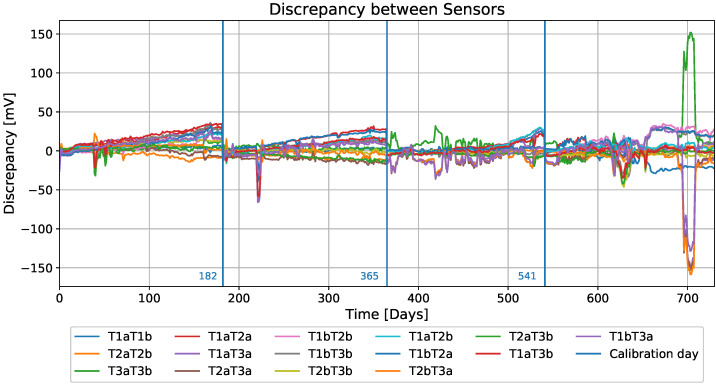
Discrepancy among the sensors’ readings, also showing calibration events on days 183, 365, and 541.

For the purposes of this work, the average data per day were used, providing a total of 731 days of data. The discrepancy between each pair of sensor readings was also calculated. In total, the discrepancy records from six sensors generated 15 records of a new dataset with a length of 731 samples. Three of these fifteen columns are discrepancies between the values of the sensors of the same type [T1a-T1b, T2a-T2b, T3a-T3b]. The raw data can be seen in Figure 7. The annotation T1a represents sensors with type 1 and in row a and T1aT2a represents the discrepancy between sensors T1a and T2a. The measured drifts on days 183, 366, and 542 were used to manually recalibrate the sensors, which means approximately one calibration every six months, resulting in four separate periods of data. In Figure 8, these days of recalibration are marked with a blue vertical line (three in total). The data in the periods between the calibration days were used as input for the proposed methods.

It is important to note that the manufacturer warranty of the sensors used to obtain the dataset only covered the first 365 days; after this, the manufacturer considered the sensors unreliable [14]. This can also be seen in Figure 7 and Figure 8, where the number of variations increased after 365 days (the red line in Figure 8 represents day 365). A brief examination of the dataset reveals that the discrepancy is not the same for most pairs of sensors. Even for the sensors of the same type, a slight increase can be seen for two pairs, while the increase seen for the last pair, T2aT2b, is more than significant. Since a dataset with measurements from ion-selective pH sensors is used in this work, a short working principle of pH sensors is presented in the following section.

### Ion-Selective pH Sensors

The core part of the pH sensors are ion-selective electrodes (ISEs). ISEs are the oldest class of chemical sensors and still outperform other types of sensors used in various biomedical, industrial, and environmental applications [56]. Clinical chemistry, in particular the determination of biologically relevant electrolytes in physiological fluids, is one of the most important fields of application for ISEs [57]. Unlike other analytical methods, ion-selective electrodes respond to ion activity rather than a concentration. The pH probe contains two electrodes: a sensor electrode and a reference electrode. The electrodes are in the form of glass tubes, one containing a pH 7 buffer and the other a saturated potassium chloride solution. A silver wire coated with silver chloride is immersed in the pH 7 buffer in the bulb. When the probe is immersed in a solution to measure pH, hydrogen ions accumulate around the bulb and replace the metal ions from the bulb. This ion exchange creates an electrical flow that is measured and converted into respective pH values [58]. The pH of a measured solution is directly related to the ratio of hydrogen ions [H^+^] and hydroxyl ions [OH^−^] concentration. A solution with more hydrogen ions [H^+^] is acidic and has a low pH (below 7) value, while solutions with more hydroxyl ions [OH^−^] are alkaline, which results in a high pH value (above 7). The values in the selected dataset represent the measured voltage between the electrodes in mV.

## 5. Discrepancy Model Estimation

As explained in Section 2.3, the discrepancy dynamics can be largely explained/modeled with simple linear or exponential models. Given this information and the fact that studies have shown that classical time series models have competitive performance compared to more complex/advanced ML methods (see Section 4), several simple models are used here to derive an estimate of the discrepancy dynamics. The models considered in this study are described in the following sections.

### 5.1. Linear Regression

Linear regression is a linear approach to modeling the relationship between a scalar response (or dependent variable) and one or more explanatory variables (or independent variables). The case of a single explanatory variable is called simple linear regression [59]. The simple linear regression model is represented by Equation (1):

(1) $$y=\beta_{0}+\beta_{1}x+\varepsilon$$

where the intercept $\beta_{0}$ and the slope $\beta_{1}$ are unknown constants, and ε is a random error component. The estimations of $\beta_{0}$ and $\beta_{1}$ are performed by using the least squares method. This means estimating $\beta_{0}$ and $\beta_{1}$ so that the sum of the squares of the difference between the observations and the estimation is minimized [59].

### 5.2. Exponential Regression

Exponential regression is presented in Equation (2):

(2) $$y=\alpha_{0}e^{\alpha_{1}x}$$

where the intercept $\alpha_{0}$ and the slope $\alpha_{1}$ are unknown constants that are estimated such that the sum of the squares of the differences between the observations and the estimation is minimized.

### 5.3. Polynomial Regression

Polynomial regression is a form of linear regression known as a special case of multiple linear regression that is used to estimate the relationship as an nth degree polynomial. Polynomial regression is sensitive to outliers, so the presence of one or two outliers can badly affect performance. Therefore, filtering is required when using polynomial regression. The general polynomial equation is given by Equation (3), where $\beta_{0},\;\beta_{1},\;\beta_{2},...\beta_{n}$ are the coefficients of the equation and *n* represents the degree of the polynomial.

(3) $$y=\beta_{0}+\beta_{1}x+\beta_{2}x^{2}+\beta_{3}x^{3}+...+\beta_{n}x^{n}$$

In this work, we used quadratic (n=2), cubic (n=3), and quartic (n=4) polynomial regression. The coefficients $\beta_{0},\;\beta_{1},\;\beta_{2},...\beta_{n}$ are estimated such that the sum of the squares of the differences between the observations and the estimation are minimized.

### 5.4. Holt’s Linear Trend Method

Unlike the simple exponential smoothing method [37], extended exponential smoothing (or Holt’s linear trend method) allows data forecasting and identification of a trend. It is important to note that two types of forecasting equations can be used: additive and multiplicative. The recursive forecasting equations for the additive model are given by Equation (4), while the multiplicative variant is given by Equation (5). In the literature, the multiplicative models are also sometimes referred to as exponential models.

(4) $$Additive:{\hat{y}}_{t+h|t}=l_{t}+hb_{t}$$

(5) $$Multiplicative:{\hat{y}}_{t+h|t}=l_{t}+{b_{t}}^{h}$$

The $l_{t}$ parameter, calculated by Equation (6), is known as the level updater, as it updates the level of the current time step based on the previous level estimate. The parameter α is known as the smoothing parameter for the level.

(6) $$Level:l_{t}=\alpha y_{t}+\left(1-\alpha \right)\left(l_{t-1}+b_{t-1}\right)$$

The $b_{t}$ parameter is the trend updater, as it updates the trend at the current time.

(7) $$Trend:b_{t}=\left(\beta \right)\left(l_{t}-l_{t-1}\right)+\left(1-{\left(\beta \right)}^{*}\right)b_{t-1}$$

The coefficient β presents the smoothing parameter for trend. In this work, both models were used.

### 5.5. Modeling

Seven different models (for a detailed description please refer to the previous section) were used to estimate the discrepancy behavior: (1) linear regression, (2) exponential regression, (3) second-degree polynomial regression, (4) third-degree polynomial regression, (5) fourth-degree polynomial regression, (6) Holt linear model with the additive trend, and (7) Holt linear model with the multiplicative trend.

The dataset [49] we used contains 15 records of discrepancies. Each record has four subdatasets determined by four time periods between calibration days (see Figure 8).

**Figure 9:**
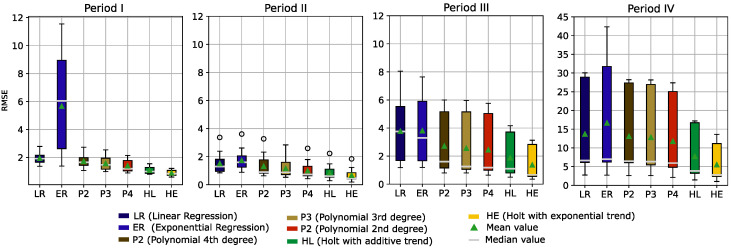
Boxplots of the root mean square error (RMSE) calculated for the trained models and categorized into four data periods.

Each of these subdatasets was used to fit the seven models. To compare the models, the root mean square error (RMSE) between the observed and fitted values for each estimation was calculated. This resulted in 15 RMSEs per model and period. Boxplots of the resulting RSME across all four periods are presented in Figure 9.

Before training the models, the data were processed with a Hampel filter and moving average filter (window = 7 days) to remove outliers and obtain smoother data.

In Figure 9, we can see that during the warranty period of the sensors (i.e., period I and period II), the RMSE values are clearly smaller. This was expected due to the higher variance and instability of the sensor readings in the last two periods. For period I (days = [0, 182]) and period II (days = [182, 356]), exponential regression gave the highest RMSE values, while the other methods showed relatively good results, with the Holt methods performing best. In period III (days = [356, 541]), RMSE values appear to increase for all models. In period IV (days = [541, 730]), the RMSE values across all models are very high compared to the previous periods.

When considering all models across all periods, the Holt method with a linear trend and the Holt method with an exponential trend show the best performance (i.e., minimizing RMSE). Therefore, these two models were selected and used in the predictive application presented in this study.

## 6. Trend Detection and Data Preprocessing

As described in Section 3, the discrepancy between the sensor readings may increase/decrease or remain stationary for a period of time. Such changes (increase or decrease) in the data can be made by detected by detecting a trend in the data. To detect trends in time series data, several groups of tests, of which the statistical tests are the most commonly used ones, are available [60].

There are two main groups of statistical tests:

- Slope-based tests, whereby the least squares linear regression method is the most commonly used.
- Rank-based tests, such as the Mann–Kendall test.

These tests have been referred to in several studies as the most commonly used tests for hydrological data, meteorological data, and climatic time series data [60,61,62].

To detect a trend in the data, it is necessary to develop a hypothesis that needs to be conducted [62]. The following pair of hypotheses were tested:

- The null hypothesis: No trend can be detected.
- The alternative hypothesis: A trend can be detected.

### 6.1. Linear Regression Trend Test

The linear regression (LR) test is a parametric test which is used to describe the presence of a linear trend in a time series. The test is applied to the slope of the regression line given by Equation (1) and is explained in Section 5.1.

### 6.2. Mann–Kendall Trend Test

The Mann–Kendall trend test [63,64] (sometimes called the MK test) is used to analyze time series data to identify consistently increasing or decreasing trends. It is a non-parametric test, which means it works for all distributions (i.e., data do not have to meet the assumption of normality), but the data should have no serial correlation. The core of MK test analysis is the sign of the difference between later-measured data and earlier-measured data, where the number of sign is counted. If the number of negative/positive signs is increasing constantly it means that a downward/upward trend is present.

### 6.3. Results

The explained trend detection methods were applied to the raw data (without processing and filtering) of the pH dataset obtained by Ohmura et al. [14]. For each sensor pair, a trend and trend rate (mV/day) were calculated and are presented in Table 2. The rate for the LR test presents the slope parameter of the filled linear function, while the rate for MK test is the Sen’s slope [65] used within the test. For all 15 pairs, both methods could be applied to detect an increasing or decreasing trend in the data. The difference between methods can be observed by examining the calculated trend’s rate. The calculated rates could be further used to form a line that estimates the data behavior. To compare the method performance, the RMSE between the data and estimation values was calculated; the results are shown in Table 2. In general, lines estimated by linear regression coefficients have a slightly better RMSE value than lines estimated by the MK test.

### 6.4. Iterative Trend Detection

**Figure 10:**
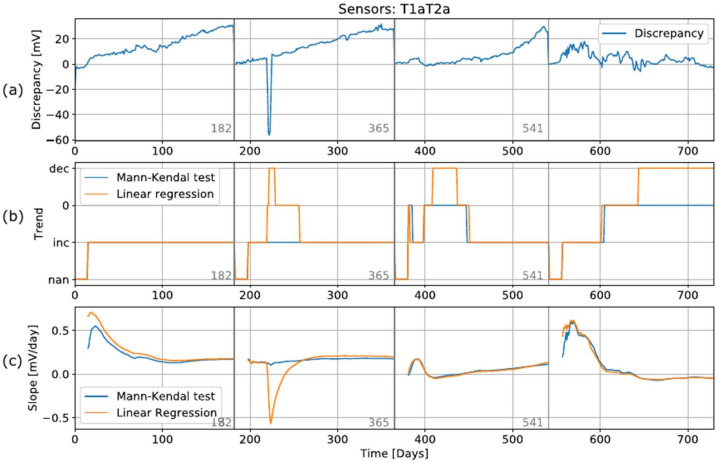
Iterative process of trend detection with raw dataset. The graphs show (**a**) raw data, (**b**) trend detection, and (**c**) slope calculated by trend detection methods.

As explained in Section 3.2, trend detection is the second step in the algorithm. The rest of the algorithm (detection and prediction) is performed only when the trend detection method detects a trend in the discrepancy. To demonstrate how this method would be applied in the algorithm, a single discrepancy record between sensors T1a and T2a was isolated. This record shows different behavioral characteristics for the different periods: linear for the first two periods and exponential for the third period (see Figure 10). In order to obtain a scenario that approximated a real environment, a new empty dataset was created. This new dataset contained the first 25 values from the original dataset, and then samples were iteratively, one by one, added. Since we had already optimized the dataset by averaging the data per day, the trend detection methods were applied in each step. The results of the trend detection are shown in Figure 10, as well as the slope of the detected trend for each cycle. Each time the calibration was performed, a new empty dataset was created, and the iteration process started again.

For the first period (days 0–183), both methods could be used to simultaneously find an increase in discrepancy. The difference between the methods can be seen by examining the slope in the third graph in Figure 10, where the MK test has a slightly lower slope. After 150 days, both tests detected the same slope. In the second period, an anomaly occurred in the measurements of one of the sensors, which resulted in an outlier on day 220. The linear detection method could be used to detect an increasing trend, then a decreasing trend after the outlier, and then a lack of trend for a period of about 30 days, followed by an increasing trend again. The MK method, on the other hand, did not reveal any such changes in the detection results. These changes can also be seen in the slope calculations, where applying the LR method showed a sudden change in the negative slope, while applying the MK method again revealed no significant changes. Therefore, we conclude that the LR method is sensitive to outliers, which can lead to incorrect conclusions, while the MK method is robust with regard to such anomalies. In the third period, applying the LR method revealed a decreasing trend, while applying the MK method did not. In addition, a small anomaly observed between day 380 and day 400 resulted in the LR method detecting a decreasing trend, while MK did not.

In the last period, the sensors showed a great deal of of instability in the measurements. Both methods could be used to detect an increasing trend up until the 600th day. A short period followed, where no trend was detected. On the 640th day, applying the LR method enabled the detection of a decreasing trend up until the end, while applying the MK method revealed no such trend.

### 6.5. Data Preprocessing

**Figure 11:**
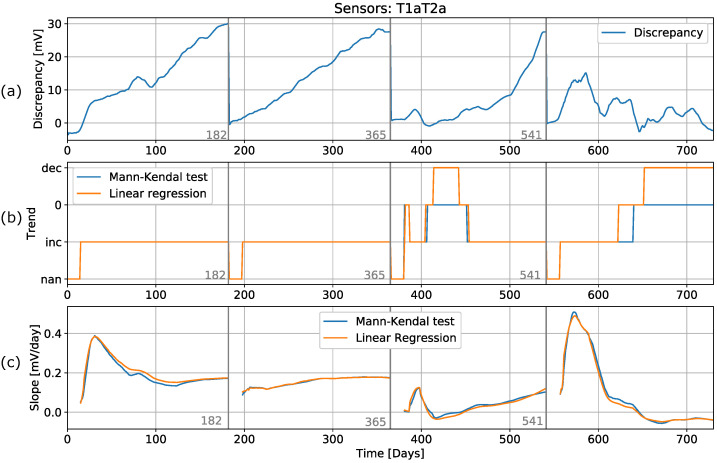
Recursive process of trend detection with filtered (Hampel filter) and averaged (window = ten) data. The graphs show (**a**) processed data (**b**), trend detection, and (**c**) the slope calculated when applying trend detection methods.

As we mentioned earlier, the first step in data preprocessing is resampling. The original dataset had a sampling frequency of 1 s. In our approach, we calculated the average per day and used this average as input for our algorithm (see Section 4). To avoid problems with outliers and noisy data, we also introduced two additional filtering methods. First, a Hampel filter was used to clean the data and remove outliers [44,45]. The Hampel filter has satisfactory estimation performance when outliers are present. The filter replaces the central value in the data window with the median if it is far enough from the median to be considered an outlier. Therefore, this is sometimes referred to as an extended median filter. The filtered data still showed the presence of noise that can affect trend detection. Since the median filter is already included in the Hampel filter, the moving average filter (window = 7) was used to further smooth the original data and to capture the main trends without the minor fluctuations. The results of applying the detection methods are shown in Figure 11. In the first period, the results from applying both trend detection methods are almost identical, as was the case for the unfiltered data. In the second period, the LR trend detection method is shown to give better results than when applied to unfiltered data. This is due to the filtering of the outlier at day 220. The MK method application provided the same results with and without filtering. In the third and fourth periods, the trend detection remained the same as for the unfiltered data. We conclude that the MK method is more robust regarding anomalies in the data but, after processing (filtering) of the data, the application of both methods gave identical results. This shows that a degradation process in the sensors can be detected with a simple trend detection, and the engineers in charge can be instructed to perform sensor maintenance.

## 7. Detection and Prediction

In Section 5.5, we showed that the discrepancy behavior can be modeled with different models and that Holt’s methods have the best performance. Therefore, these models were subsequently used to predict the behavior of the discrepancy in the future and to estimate when the discrepancy would reach the maximum allowable value, the threshold.

The most recent samples of the filtered discrepancy signal were split into training samples and holdout samples (the duration of the holdout samples is 14 days, which corresponds to 14 data points). Training samples were used to fit two models: Holt with linear trend (HL) and Holt with exponential trend (HE). These models were then used to predict the behavior of the discrepancy for the period of the holdout data samples (i.e., the next subsequent 14 days). The RMSE between the predicted values and the holdout samples was then calculated, and the values were compared. The model with better performance (i.e., with lower RMSE) was selected, and its long-term prediction (90 days) was chosen for the failure event prediction.

In order to evaluate the failure event prediction performance, we introduced the failure timepoint prediction error defined in Equation (8). We resampled the dataset to obtain one data point per day; therefore, we refer to days below, but each day corresponds to one data point.

(8) $$Error=DTF_{real}-DTF_{predicted}$$

The error represents the difference between the actual number of days to failure $DTF_{real}$ and the predicted number of days to failure $DTF_{predict}$. The values are also illustrated in Figure 5. In addition to the error itself, this measure also indicates whether the failure is predicted to occur before or after the actual failure. If the error is positive, it means that the failure was predicted before it occurred. In this case, the algorithm will suggest replacement of the sensor before its actual failure. To some extent, this situation can be tolerated, because replacing the sensor is still cheaper than allowing the failure to occur. On the other hand, if the error is negative, it means that the system will fail on the predicted failure timepoint.

The $DTF_{real}$ value used in Equation (8) is the day on which the discrepancy will reach the threshold. This value is programmatically extracted from the dataset. Therefore, the $DTF_{real}$ values used in the evaluation depended on the discrepancy threshold.

The $DTF_{predicted}$ value was calculated using the best fitting model among the two trained models. The predicted data were searched to identify the threshold index, where the predicted data are equal to the threshold. At this point, the failure timepoint prediction error could be calculated, which is visually explained in Figure 5. The algorithm predicts failures only for the next 90 days; therefore, failures that occur further in the future than 90 days are not considered (no failure event is predicted in this case).

**Figure 12:**
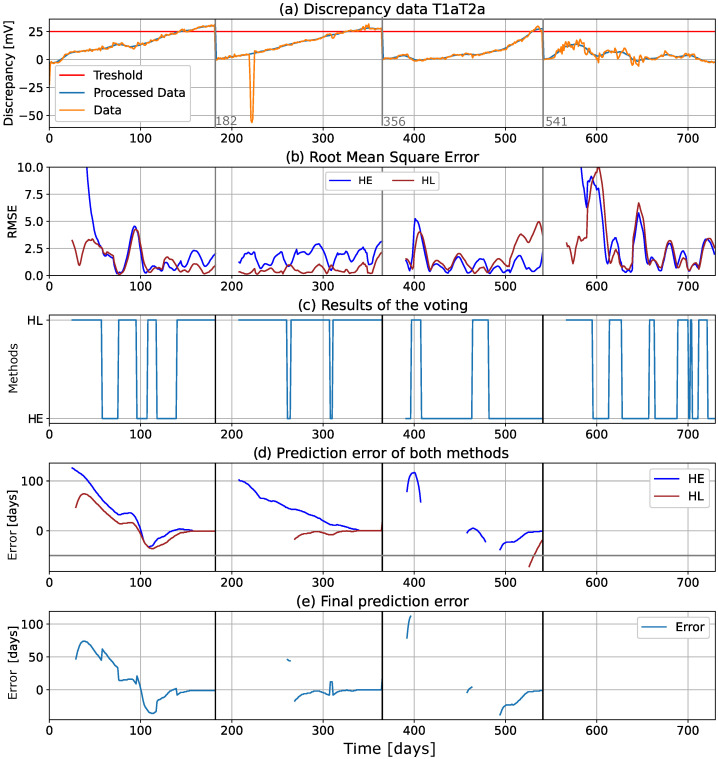
Example of the algorithm’s calculations for data record “T1aT2a”: (**a**) discrepancy data with threshold, (**b**) RMSE values for holdout values, (**c**) results of the voting, (**d**) prediction errors of both methods, and (**e**) final results of the algorithm based on the voting.

Figure 12 shows the computational graphs for an isolated T1aT2a record, with a threshold of 25 mV (highlighted in red in the graph). This dataset was chosen as an example because it displayed two different behaviors during different time periods. In the warranty period ([0, 365 days]), the discrepancy showed a linear behavior, while in period III, it showed an exponential increase. Period IV of the data showed a large variance, but no significant trend could be detected. Figure 12a shows the data. The RMSE metric of the fitting models is shown in Figure 12b, and the model selection (based on the RMSE of the 14 holdout data points shown in Figure 12b) is shown in Figure 12c, where the HL method is clearly better in the first two periods, while HE is better in period III. The failure timepoint prediction error (defined by Equation (8)) is shown in Figure 12d, while Figure 12e shows the final error after voting, which corresponds to the best fitting model.

In the first three periods, it can be seen that the prediction error decreases as the size of the training data increases (i.e., as the size of the training dataset increases), the error decreases, and the prediction becomes more accurate. The fitting models are used to predict, at each time step or data point, the next 90 days of the discrepancy signal. If no value is shown in Figure 12d,e, it means that the threshold will not be reached during the next 90 days. This happens in period III, where some values are missing, and throughout the whole of period IV, where no values are shown, because the predicted discrepancy never reached the threshold. This finding is confirmed by examining the data in Figure 12a, where it can be seen that the discrepancy clearly never reaches the threshold in period IV.

### 7.1. Performances of the Prediction

**Figure 13:**
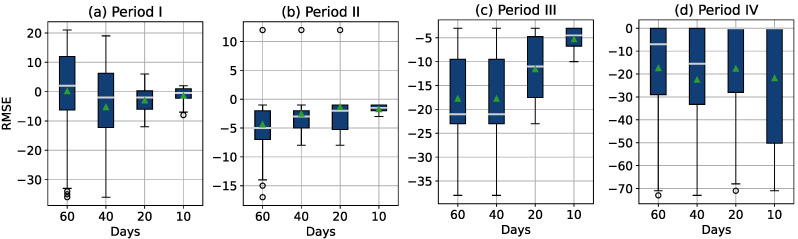
Prediction error for the last 60, 40, 20, and 10 days before threshold is reached, where green triangles represent mean values, for (**a**) period I ([0, 182] days), (**b**) period II ([183, 356] days), (**c**) period III ([356, 541] days), and (**d**) period IV ([542, 730] days).

To evaluate the accuracy of the prediction performance, the prediction errors for the last two months before the thresholds were reached are presented in the form of a boxplot in Figure 13. Four boxplots were constructed for each data period, and each boxplot shows the failure timepoint estimation error 60, 40, 20, and 10 days before the threshold is reached. For the first 365 days (period I and period II), the prediction error and the mean and median values are in the range of ±5 days and it is closer to zero as the threshold approaches. In period III, the mean and median values can be found in the range of [−23,−5] days. In period IV, the range of the failure timepoint estimation error is much higher, but this was to be expected due to the rapid variance in discrepancy signals in this period (note also that the sensors are no longer covered by warranty in this period).

### 7.2. Accuracy of the Detection

In order to quantify the accuracy of our proposed failure event prediction method, a confusion matrix was constructed. The basic terms required for the confusion matrix were defined, depending on the prediction and the occurrence of the failure event (i.e., the discrepancy reaches the threshold) for each data point and its next 90 days (data points), as follows:

- True positive (TP): The failure event is predicted to occur, and it actually occurs.
- True negative (TN): The failure event is not predicted to occur, and it does not occur.
- False positive (FP): The failure event is predicted to occur, but it does not occur.
- False negative (FN): The event is not predicted to occur, but it does occur.

**Figure 14:**
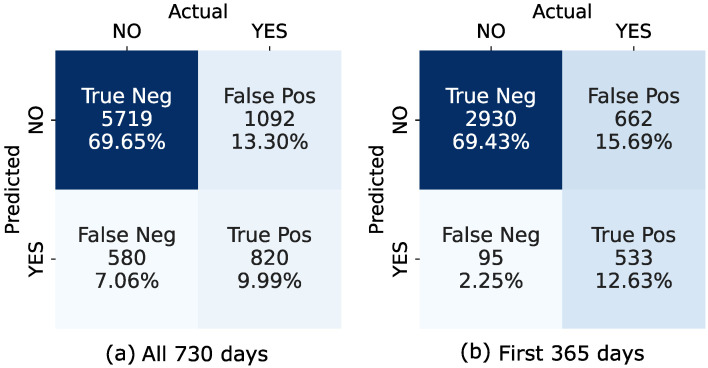
Confusion matrices for event detection, where the presented approach is applied to (**a**) all days of the dataset ([0, 730] days); (**b**) days of the warranty period of the sensors ([0, 365] days).

Figure 14b shows results for the period during which the sensors were still under the manufacturer warranty, while Figure 14a shows results for all days. The accuracy metrics calculated from confusion matrices are shown in Table 3. In addition to the rates for the warranty and the entire period, the rates for the individual four periods are also shown.

When examining the data and the confusion matrix results, we noted that the main reason that a false positive occurred was that some records were close to reaching the threshold but did not because of the sensor recalibration day. On some occasions, oscillatory noise (or seasonality) remained as small deviations in the filtered discrepancy and caused the algorithm to produce a false positive result.

On the other hand, false negative (FN) results occurred when the trend in the discrepancy increased very rapidly, as was the case in the third presented example shown in Figure 12. In this case, our approach enabled us to estimate a trend with a delay (some data points later), and to predict a failure timepoint further in the future than 90 days. Using the Holt method with the exponential trend provided the best results in this situation, but false negatives do still occur in some cases. This behavior is especially evident in periods III and IV of the discrepancy data, where the sensors were no longer under the manufacturer’s warranty.

The values in Table 3 show that our method has an accuracy rate of 0.8 (80%) correct predictions for the whole dataset (730 days) and an accuracy rate of 0.82 (82%) for the period when the sensors are under warranty. The parameter AccuracyII=(TP+TN+FP)/SUM presents the accuracy of the algorithm if the false positives (detection of the failure when failure will not occur in the next 90 days) are defined as acceptable results. If we examine the individual periods, we can see that the accuracy in period I is lower compared to that in periods II and III. This is due to the fact that we had the highest number of false positive predictions in period I, where the data were close to reaching the threshold but did not, thanks to sensor recalibration day. In period IV, the detection accuracy was good, with a value of 0.69 (69%), even though the sensor data quality is significantly worse than in the previous periods.

## 8. Generalization

In this section, we discuss whether the proposed approach is generalizable to other sensor types and applications. To this end, we summarize the assumptions underlying our method concerning sensors and sensor data and examine whether these assumptions can be applied to/hold in the context of other sensor types. As our method was developed to detect drift faults, the end of the section addresses ability of the presented method to handle other common faults in sensors as well as intermittent faults.

Due to the general shortage of publicly available datasets with data from redundant safety architectures (e.g., 1oo2), the algorithm was only tested on a limited number of redundant pH sensors. For this reason, we would like to evaluate the advantages and disadvantages of our approach with other sensor types in future work.

The key requirement for our approach is related to hardware: A one-out-of-two redundant sensor safety architecture needs to be implemented, where the measurements from two identical sensors in the same environment are compared. If this was not possible, the discrepancy analysis (i.e., our approach) could not be applied.

Although the discrepancy signal depends on the individual drift of the two sensors, there were no special requirements regarding their individual drift behavior. The only requirement when taking this approach is that the redundant sensors (after calibration, if necessary) show similar behavioral models with small deviations. This is the case in many applications (e.g., certain gas sensors [9], pressure sensors [10,11,12], pH sensors [13], or humidity sensors [14,15]), but this condition is not met in every case and needs to be carefully investigated.

The first step of the proposed approach is the data preprocessing step. A two-step filter is implemented (initially, a Hampel filter is used to reject outliers and then a moving average filter is used to smooth the signal) to increase the accuracy and robustness of the trend estimation. The two-step filter ensures the robustness of the algorithm regarding various anomalies, such as outliers, missing data points, or high-frequency noise in the data. Thus, these anomalies in the data do not cause the algorithm to perform more poorly. However, the filters can be adjusted if they are not effective enough. The Hampel filter is similar to a median filter. Therefore, one must be careful when using it, because the filter use can affect analyses that use derived or integrated sensor data, that is, its use eliminates oscillations in the data that may be important.

Before our approach provided any output, a certain amount of data needed to be buffered, both for the two-step filter and for trend estimation. For some applications where the initial data points are important, a different approach may need to be taken for this purpose. For example, classical discrepancy analysis works with a threshold and requires only one data point in the simplest case.

Another aspect that needed to be considered in our approach was the delayed trend estimation when the discrepancy signal did not grow linearly (e.g., when the value of the second derivative was high). In this case, although the algorithm can be used to detect the increase in discrepancy and to estimate the timepoint when failure should occur, the actual failure will occur before the estimated timepoint. This does not allow maintenance to be effectively scheduled on the system where the redundant sensor is installed. This effect was partially aggravated by our use of a two-step filter, which was required for outlier suppression and smoothing. One might think that choosing between a second-order polynomial and the linear model for the fitting model would alleviate this problem. However, in our work, we studied the use of multiple fitting models and found that the comparison of the linear Holt model with the Holt exponential model based on the RMSE fitting error provided the best results for estimating when failure would occur.

If the discrepancy does not behave uniformly (i.e., shows different behavior in different time periods), which in some cases can also be referred to as low-frequency noise or seasonality, the algorithm may produce many false positives. This behavior is illustrated in Figure 12 in period IV, where the algorithm produces the most false positives at the beginning due to the increasing discrepancy. However, this behavior was not observed in the periods when the sensors were under warranty.

The main focus of the presented method is to detect the increase in discrepancy between sensors and to predict a point in time when discrepancy reaches the threshold, where it is considered a fault. An increase in discrepancy indicates that at least one of the sensors is drifting. The method cannot detect an individual drift in a sensor if both sensors have identical drift dynamics. In addition to drift fault, sensors may exhibit other types of faults, such as precision degradation (increased noise and outliers), bias, and stuck at faults [66]. Since our first step is data filtering, detecting increased noise and outliers is impossible because the filters smooth the signal and remove outliers. If a bias is present in one of the sensors, the trend detection will show a constant difference between the redundant signals, indicating that one of the sensors is biased and calibration is needed. Errors such as “stuck at” are detected by the inherent property of classical discrepancy analysis, and the system enters a safe state to prevent further damage. Since discrepancy between the sensors constitutes the basis of the method, a fault occurring in both sensors at the same time with the same fault characteristics will not be detected by our method.

The method proposed in this article was initially designed to forecast permanent faults, but the concept can also be used to detect intermittent faults if the intervals of occurrence or the causes of the intermittent faults are known. If this is the case, the data points from these intervals can be separated and used as input to our method to detect changes in drift.

## 9. Conclusions and Outlook

In this paper, we propose a novel fault prognosis algorithm based on the discrepancy signal for the safety architecture with 1oo2 redundant sensors.

During our study, we concluded that there is a shortage of publicly available datasets for research in this context. Therefore, we selected the most appropriate several publicly available datasets and used them for our research. This selection led us to focus our research on pH sensors that are affected by aging, which resulted in increasing noise and a drift in the readings over time in the dataset.

Our fault prognosis algorithm provided us with a key advantage over classical discrepancy analysis in that it allowed us to predict when the sensor system would fail. This is the point in time when the discrepancy signal is predicted to reach the threshold and trigger the safe state. Based on the entire dataset, which contains a total of 15 discrepancy records, our results indicated that the timepoint of the failure event can be accurately predicted 82% of the time and, in some specific configurations, the accuracy can even increase up to 97%. The prediction of the failure timepoint was further estimated in days (with a sampling rate of one data point per day) and its accuracy was evaluated in terms of failure timepoint prediction error. While the pH sensors were under warranty, the achieved timepoint prediction accuracy for all 15 deviation datasets was good enough to predict the failure timepoint with a comparatively low error as compared to the actual real days until failure, which, in any case, would have left enough time for maintenance to be performed on the sensor system.

The ability to predict the failure timepoint was achieved by prudently applying the following processing steps to the raw discrepancy signal: (1) a two-step filter to reject outliers and smooth the signal; (2) a trend detection test followed by estimation of the trend by fitting two models to the most recent data points (excluding the holdout record); (3) a selection of the best fitting model based on the RMSE for the holdout record data points (which are the most recent filtered discrepancy data points). In the corresponding sections of this paper, we presented the results of analyzing the accuracy achieved with each of these processing steps and explained the reasoning behind our design decisions.

Regarding our analysis, we have explained the failure cases of our proposed algorithm, which may form starting points for future work. For example, oscillating discrepancy signals at some frequencies present problems for our two-step filter and trend estimation method. Alternatives to the two-step filter should be studied not only for this reason but also because they contribute to a delayed estimation of the trend of the discrepancy signal. Improvements in this area would lead to higher accuracy, both in terms of better prediction of failure events and lower error variance in predicting the failure timepoint. In addition, we would like to assess the advantages and disadvantages of our approach for sensor types other than pH sensors. Another question for follow-up work concerns the questions of how well the method performs when different types of errors are present (in both sensors), and how the method can be improved to detect/predict as many errors as possible. Finally, we would like to emphasize that more publicly available datasets with redundant sensor data are clearly needed for this and similar research directions.

## Figures and Tables

**Table 1:** Publicly available datasets with redundant sensors that were analyzed.

Dataset	Type	Redundant	Units	Time Span	Continues	Sample Rate	Samples	Reference
MOX Gas_1	Metal Oxide	Yes	16	36 months	No	Not Defined	13,910	[46,52]
MOX Gas_2	Metal Oxide	Yes	16	12 h	Yes	100 Hz	4,178,504	[50]
MOX Gas_3	Metal Oxide	Yes	10	600 s	Yes	100 Hz	640	[53,54]
MOX Gas_4	Metal Oxide	Yes	8	2 months	Yes	Not Defined	919,438	[55]
MOX Gas_5	Metal Oxide	Yes	14	17 days	Yes	3.5 Hz	4,095,000	[56,57]
PhSensors	pH	Yes	10	731 days	Yes	1 Hz	59,537,757	[14,49]

**Table 2:** Results of trend detection methods with calculated trend rates and RMSE values for each of the sensor pairs.

Pair	Mann–Kendall			Linear Regression		
	Trend	Rate	RMSE	Trend	Rate	RMSE
T1aT1b	inc	0.025674	7.719319	inc	0.036238	6.752130
T2aT2b	dec	0.017322	4.748087	dec	0.015198	4.617770
T3aT3b	inc	0.005690	12.374313	inc	0.019710	11.952504
T1aT2a	inc	0.135080	5.916490	inc	0.131836	5.878657
T1aT3a	inc	0.110462	14.651749	inc	0.088871	13.520241
T2aT3a	dec	0.034774	10.505421	dec	0.042965	10.317161
T1bT2b	inc	0.078881	4.291423	inc	0.080400	4.271486
T1bT3b	inc	0.071403	7.305578	inc	0.072342	7.179803
T2bT3b	dec	0.009431	4.719706	dec	0.008057	4.574653
T1aT2b	inc	0.113613	4.885449	inc	0.116638	4.832450
T1bT2a	inc	0.094930	5.520764	inc	0.095598	5.502349
T2bT3a	dec	0.007732	12.349013	dec	0.027767	11.229485
T2aT3b	dec	0.026685	3.772427	dec	0.023256	3.677405
T1aT3b	inc	0.109684	4.917770	inc	0.108580	4.856540
T1bT3a	inc	0.066751	11.328040	inc	0.052633	10.684941

**Table 3:** Metrics calculated from confusion matrices.

	730 Days	365 Days	Period I	Period II	Period III	Period IV
Accuracy (TP + TN)/SUM	0.8	0.82	0.78	0.86	0.85	0.7
Accuracy II (TP + TN + FP)/SUM	0.93	0.98	0.97	0.98	0.91	0.82
Error (FP + FN)/SUM	0.2	0.18	0.22	0.14	0.15	0.3
Sensitivity TP/(TP + FN)	0.59	0.85	0.87	0.8	0.45	0.33
Specificity TN/(TN + FP)	0.84	0.82	0.76	0.86	0.91	0.83
FP Rate FP/(TN + FP)	0.16	0.18	0.24	0.14	0.09	0.17
FN Rate FN/(TP + FN)	0.41	0.15	0.13	0.2	0.55	0.67

## Data Availability

In this work we analyzed sensors measurement from the publicly available datasets accessible in [52,53,67], while the pH sensor dataset used for detection and prediction is accessible in [49].
